# Discordance between 'actual' and 'scheduled' check-in times at a heart failure clinic

**DOI:** 10.1371/journal.pone.0187849

**Published:** 2017-11-14

**Authors:** Eiran Z. Gorodeski, Emer Joyce, Benjamin T. Gandesbery, Eugene H. Blackstone, David O. Taylor, W. H. Wilson Tang, Randall C. Starling, Rory Hachamovitch

**Affiliations:** 1 Section of Heart Failure and Cardiac Transplantation, Tomsich Family Department of Cardiovascular Medicine, Heart and Vascular Institute, Cleveland Clinic, Cleveland, Ohio, United States of America; 2 Center for Connected Care, Medical Operations, Cleveland Clinic, Cleveland, Ohio, United States of America; 3 Case Western Reserve University School of Medicine, Cleveland, Ohio, United States of America; 4 Department of Quantitative Health Sciences, Cleveland Clinic, Cleveland, Ohio, United States of America; 5 Section of Cardiovascular Imaging, Tomsich Family Department of Cardiovascular Medicine, Heart and Vascular Institute, Cleveland Clinic, Cleveland, Ohio, United States of America; University Medical Center Goettingen, GERMANY

## Abstract

**Introduction:**

A 2015 Institute Of Medicine statement “Transforming Health Care Scheduling and Access: Getting to Now”, has increased concerns regarding patient wait times. Although waiting times have been widely studied, little attention has been paid to the role of patient arrival times as a component of this phenomenon. To this end, we investigated patterns of patient arrival at scheduled ambulatory heart failure (HF) clinic appointments and studied its predictors. We hypothesized that patients are more likely to arrive later than scheduled, with progressively later arrivals later in the day.

**Methods and results:**

Using a business intelligence database we identified 6,194 unique patients that visited the Cleveland Clinic Main Campus HF clinic between January, 2015 and January, 2017. This clinic served both as a tertiary referral center and a community HF clinic. Transplant and left ventricular assist device (LVAD) visits were excluded. Punctuality was defined as the difference between ‘actual’ and ‘scheduled’ check-in times, whereby negative values (i.e., early punctuality) were patients who checked-in early. Contrary to our hypothesis, we found that patients checked-in late only a minority of the time (38% of visits). Additionally, examining punctuality by appointment hour slot we found that patients scheduled after 8AM had progressively earlier check-in times as the day progressed (P < .001 for trend). In both a Random Forest-Regression framework and linear regression models the most important risk-adjusted predictors of early punctuality were: later in the day appointment hour slot, patient having previously been to the hospital, age in the early 70s, and white race.

**Conclusions:**

Patients attending a mixed population ambulatory HF clinic check-in earlier than scheduled times, with progressive discrepant intervals throughout the day. This finding may have significant implications for provider utilization and resource planning in order to maximize clinic efficiency. The impact of elective early arrival on patient’s perceived wait times requires further study.

## Introduction

A 2015 Institute Of Medicine statement entitled “Transforming Health Care Scheduling and Access: Getting to Now”, has increased concerns regarding patient wait times. Although waiting times have been widely studied, little attention has been paid to the role of patient arrival times as a component of this phenomenon [1].

A lack of patient punctuality to a scheduled outpatient appointment, either in the patient being too early or too late, can lead a variety of undesirable consequences. A patient who shows up too early may have an extended wait time which can degrade perceived satisfaction with the visit [2]. At times providers may try to accommodate the early arriving patient at an earlier time slot [3], which can subsequently disrupt the timeliness of care for other patients who are scheduled in subsequent slots and arrive in a timely manner. On the other hand, a patient who shows up too late may have a prolonged wait time until the provider is able to see them on the same day, or may even have their appointment cancelled entirely. In both situations a lack of punctuality disrupts the flow of the outpatient clinic and creates stress for the patient and the provider. The ideal situation is minimization the absolute value of the deviation from the scheduled appointment time.

The aim of our study was to evaluate the discrepancy between ‘actual’ and ‘scheduled’ check-in times, or punctuality, at the HF clinic in Cleveland Clinic Main Campus, a large tertiary hospital in Cleveland, Ohio. We hypothesized that patients attending this clinic are more likely to arrive later than scheduled, with progressively later arrivals later in the day.

## Methods

We performed a study of consecutive patients who presented to the Cleveland Clinic Main Campus HF clinic between January, 2015 and January, 2017, using an administrative database of patient visits. This outpatient clinic served both as a tertiary referral center and a community HF clinic, and was staffed by physicians and advanced practice practitioners (APPs), including nurse practitioners and physician assistants.

Patients had to have scheduled appointments prior to arriving. Visits were scheduled for 30 or 60 minutes depending on type: new to the HF clinic and the hospital (60 minutes), new to the HF clinic but not the hospital usually representing internal consults (60 minutes), and established patients previously seen in the HF clinic (30 minutes). Clinicians were assigned templates that alternated visit types (**S1 File**), and generally worked either whole or half days (mornings ending at 1:00PM, or afternoons ending at 5:00PM). Clinicians had the flexibility to add on patients between 7:30AM at the earliest and 4:30PM at the latest, as well as to double book. Upon arrival to the lobby outside the HF clinic, located on the 3^rd^ floor of the hospital, patients were checked-in digitally by a medical secretary.

The cohort was limited to the first visit of each unique patient during the study’s time period (January, 2015 and January, 2017). The cohort was further limited to those patients scheduled to be seen by a single provider in the HF clinic on the day of visit. Heart transplant, left ventricular assist device (LVAD), social work, and research visits were excluded. The study was approved by Institutional Review Board of the Cleveland Clinic.

### Study variables

Demographic and visit-specific variables were obtained from Cleveland Clinic’s hospital business intelligence database. There were no missing data. ‘Scheduled’ check-in times and ‘actual’ check-in times were recorded automatically via Cleveland Clinic’s appointment system. Weather-related variables were obtained from the National Oceanic and Atmospheric Administration’s National Centers for Environmental Information website (www.ncei.noaa.gov), and merged in by calendar date. These included temperature (°F) on day of visit, average wind speed (mph), rain fall (inch), snow fall (inch), snow depth (inch), presence of fog (yes/no), and presence of haze (yes/no).

### End point

Punctuality, a continuous variable comprised of one minute intervals, was defined as the difference between ‘actual’ and ‘scheduled’ check-in times, whereby positive values were late check-in times and negative values were early check-in times.

### Statistical analyses

Cohort characteristics were summarized with continuous variables expressed as mean and standard deviation, and categorical variables as frequencies. The Wilcoxon-Kruskal-Wallis test was used for continuous variables, the Pearson chi-square test was used for categorical variables, and the likelihood ratio chi-square test from the proportional odds model was used for ordinal variables. The distribution of punctuality was explored graphically, with point estimates expressed as mean and 95% bootstrapped confidence interval (CI).

We used random forest-regression (RF-R) and multiple linear regression methodologies to investigate the association between the independent variables and punctuality. The primary purpose of utilizing RF-R was for variable selection for linear regression modeling. A secondary purpose of RF-R methodology was to graphically explore the relation between the most predictive variables.

Random forests, a robust machine-learning statistical methodology, have been previously described in context of the medical literature by our group and others [4–7]. Briefly, RF-R allow for ranking of variables by importance, and investigation of each variable’s risk-adjusted behavior, using decision tree methods that are independent of parametric assumptions, and inherently account for interactions and outliers. We constructed an RF-R framework composed of 1,000 uncorrelated decision trees grown from the 20 variables shown in **Table 1**, for the outcome punctuality. Trees in this RF-R framework, essentially a set of uncorrelated massively complex prediction algorithms, were broad and densely branched with a mean 1,272 terminal nodes.

**Table 1:** Cohort characteristics.

	Overall cohort	Checked-in early or on time	Checked-in late	P-value
	(n = 6,194)	(n = 3,845)	(n = 2,349)	
Age, range	61 (18–100)	62 (18–100)	60 (18–96)	< .001
Men, %	3,703 (60)	2,349 (61)	1,354 (58)	.007
Race, %				< .001
White	4,672 (75)	3,017 (78)	1,655 (70)	
Black	1,294 (21)	717 (19)	577 (25)	
Other	228 (4)	111 (3)	117 (5)	
Insurance, %				< .001
Medicare	3,212 (52)	2,070 (54)	1,142 (49)	
Medicaid	487 (8)	267 (7)	220 (9)	
Other	2,495 (40)	1,508 (39)	987 (42)	
Lives in same county as hospital, %	1,750 (28)	1,064 (28)	686 (29)	.2
International patient, %	186 (3)	86 (2)	100 (4)	< .001
Visit provider, %				.3
Physician	5,932 (96)	3,690 (96)	2,242 (95)	
Advanced practice provider	262 (4)	155 (4)	107 (5)	
Visit type, %				< .001
New to HF clinic and hospital	1,562 (25)	793 (21)	769 (33)	
New to HF clinic but not hospital	1,812 (29)	1,162 (30)	650 (28)	
Previously seen in HF clinic	2,820 (46)	1,890 (49)	930 (40)	
Appointment hour slot, %				< .001
7AM–8AM	402 (7)	162 (4)	240 (10)	
8AM–9AM	784 (13)	423 (11)	361 (15)	
9AM–10AM	826 (13)	478 (12)	348 (15)	
10AM–11AM	819 (13)	509 (13)	310 (13)	
11AM–12PM	901 (15)	611 (16)	290 (12)	
12PM–1PM	914 (15)	601 (16)	313 (13)	
1PM–2PM	674 (11)	448 (12)	226 (10)	
2PM–3PM	686 (11)	469 (12)	217 (9)	
3PM–5PM	188 (3)	144 (4)	44 (2)	
Total outpatient appointments in the hospital on the same day including HF clinic visit, %				< .001
One	4,577 (74)	2,778 (72)	1,799 (77)	
Two	1,048 (17)	704 (18)	344 (15)	
Three or more	569 (9)	363 (9)	206 (9)	
HF Clinic visit was first or only appointment of the day, %	4,577 (74)	2,778 (72)	1,799 (77)	< .001
Day of week, %				.04
Monday	1,114 (18)	704 (18)	410 (17)	
Tuesday	1,321 (21)	808 (21)	513 (22)	
Wednesday	1,414 (23)	891 (23)	523 (22)	
Thursday	1,277 (21)	752 (20)	525 (22)	
Friday	1,068 (17)	690 (18)	378 (16)	
Calendar year quarter, %				.6
First quarter	2,162 (35)	1,356 (35)	806 (34)	
Second quarter	1,742 (28)	1,072 (28)	670 (29)	
Third quarter	1,201 (19)	731 (19)	470 (20)	
Fourth quarter	1,089 (18)	686 (18)	403 (17)	
Weather on day of appointment				
Temperature, °F	50 (21)	49 (21)	50 (21)	.3
Average wind speed, mph	9.6 (4)	9.6 (3.8)	9.6 (3.8)	.7
Rain fall, inch	0.1 (0.2)	0.1 (0.2)	0.1 (0.2)	.9
Snow fall, inch	0.1 (0.5)	0.1 (0.5)	0.1 (0.5)	1
Snow depth, inch	1.0 (0.5)	1 (2.3)	1 (2.3)	.5
Presence of fog, %	2,835 (46)	1,781 (46)	1,054 (45)	.3
Presence of haze, %	1,235 (20)	776 (20)	459 (20)	.5

Continues variables shown as mean and standard deviation, unless otherwise noted

We identified the most important variables in the RF-R framework as those that most frequently split the branches near the tree trunks (i.e., minimal depth), averaged over the entire forest [5–7]. Minimal depth is a dimensionless statistic that measures the predictiveness of a variable in tree-based models. Smaller values for minimal depth indicate better predictiveness [8]; Just as “cream rises to the top,” the most predictive variables in random forest frameworks tend to aggregate closet to the root node (labeled level 0) of decision trees. The distribution of minimal depths (D_*v*_) across the forest was calculated, and the mean D_*v*_ was derived. This was used as a filtering threshold for identifying those variables that were informative for prediction [7].

For presentation purposes we then explored the relation between the top four variables identified by minimal depth analysis and punctuality using RF-R framework-derived partial dependence plots. These can be interpreted as the effect on the response for a one unit change in the predictor, while averaging over the effects of all other variables [9]. Additionally, we created a marginal plot visualizing the simultaneous effect of the top four variables on the outcome.

Finally, to quantitatively describe the risk-adjusted behavior of ‘appointment hour slot’, our key variable of interest, we constructed fully-adjusted multiple linear regression models.

### Computational methods

All analyses were performed with R version 3.2.3 (www.r-project.org), running off a Linux operating system, on a Dell PowerEdge R180 server. We used Wickham’s tidyr and dplyr libraries for data tidying and manipulation, lubridate [10] library for manipulation of dates and times, and ggplot2 [11] for data visualization and graphics. We used Ishwaran and Kogalur’s randomForestSRC [12] library for RF-R analyses, and Harrell’s rms and Hmisc libraries for regression analyses.

## Results

We identified 6,194 patients that met the study’s inclusion and exclusion criteria (**S1 Fig**). Of 27,654 visits to the HF clinic in the study time period of January, 2015 and January, 2017, we excluded visits of patients with heart transplants (n = 1,834) or LVADs (n = 3,674), visits with multiple providers on same day (n = 2,886), and visits with non-clinicians such as those with social worker or research staff (n = 1,493). Finally, we excluded all subsequent visits beyond the first for those patients who had >1 visit in the study period (n = 11,573).

**Fig 1:**
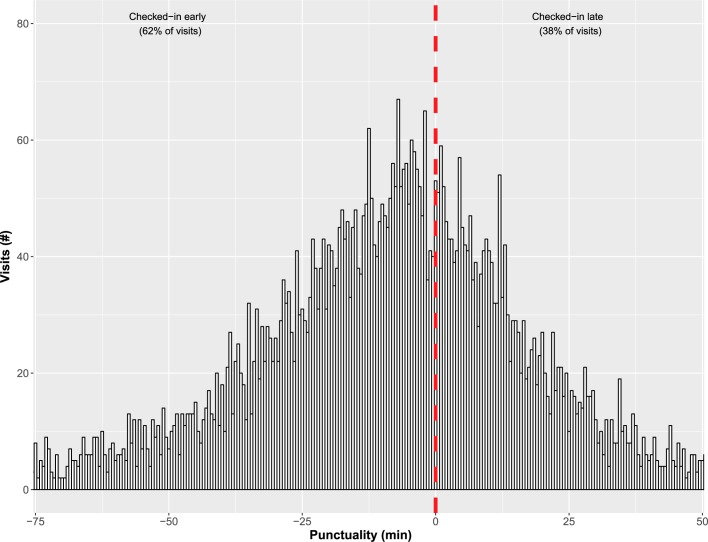
Distribution of punctuality (differences between ‘actual’ and ‘scheduled’ check-in times). Dotted line represents a visit for which a patient checked-in exactly on time (punctuality = 0).

**Fig 2:**
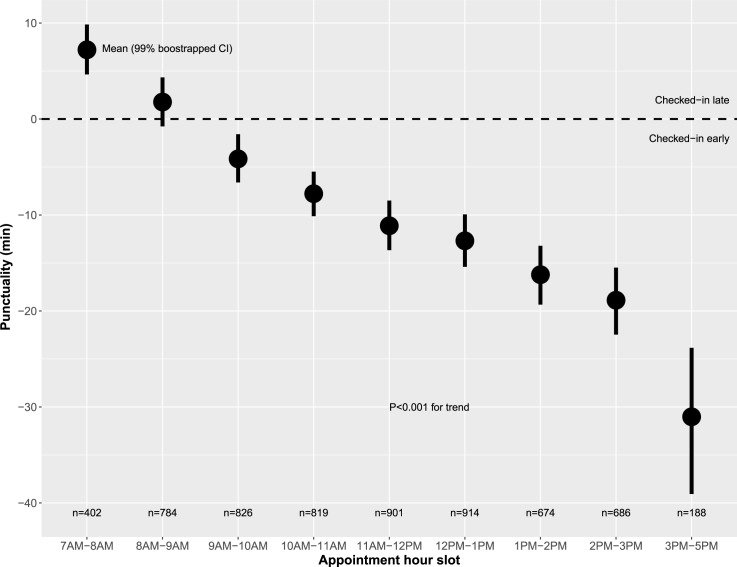
Punctuality according to appointment hour slot.

Among the 6,194 patients in the study cohort 38% checked-in late (n = 2,349 visits) (**Fig 1**). As compared to those who checked in late, patients who checked-in early/on time were (**Table 1**) proportionally older, more frequently white and male, and more frequently Medicare beneficiaries. Further, they were more likely to have previously been seen in the HF clinic. Patients tended to check-in early/on time for later appointment hour slots in the day (**Table 1**, **Fig 2**). Weather on day of appointment, including temperature, wind, rain, snow, fog, and haze, did not impact check-in time patterns in unadjusted analyses (**Table 1**).

**Fig 3:**
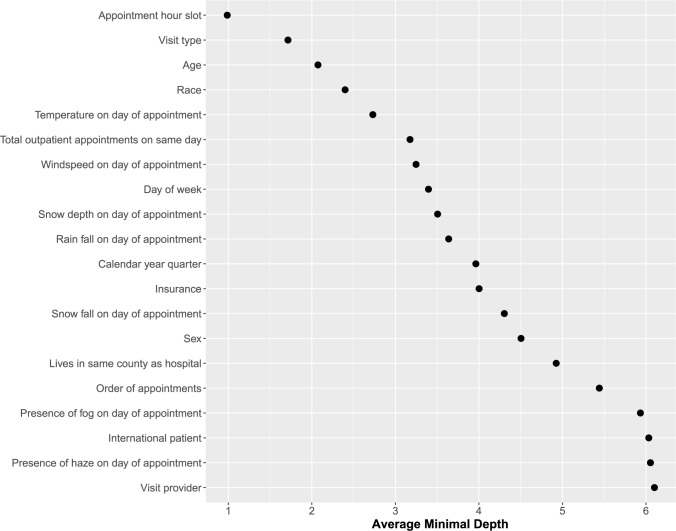
Predictiveness of punctuality, on basis of average minimal depth ranking of variables. Appointment hour slot was the most predictive variable, followed by visit type, and so forth.

**Fig 3**shows all 20 variables and plots their average minimal depth as derived from the RF-R framework. All variables exceeded a threshold value for filtering variables, therefore confirming their contribution towards predictiveness. Of these, the most predictive variables are those seen in the top left, including appointment hour slot (minimal depth = 0.99), visit type (1.7), age (2.1), race (2.4), temperature on day of appointment (2.7), and total outpatient appointments on same day (3.2), among others. We constructed two sets of fully-adjusted multiple linear regression models (**S1 Table**). In the first model appointment hour slot was modeled as a continuous variable, showing a 3.4 minute progressively earlier predicted check-in time per hour, as the day progressed. In the second model appointment hour slot was modeled as an ordinal variable, showing that as compared to the earliest appointment hour slot of the day those visits scheduled for the latest slots in the day were estimated to check-in approximately 35 minutes earlier.

**Fig 4:**
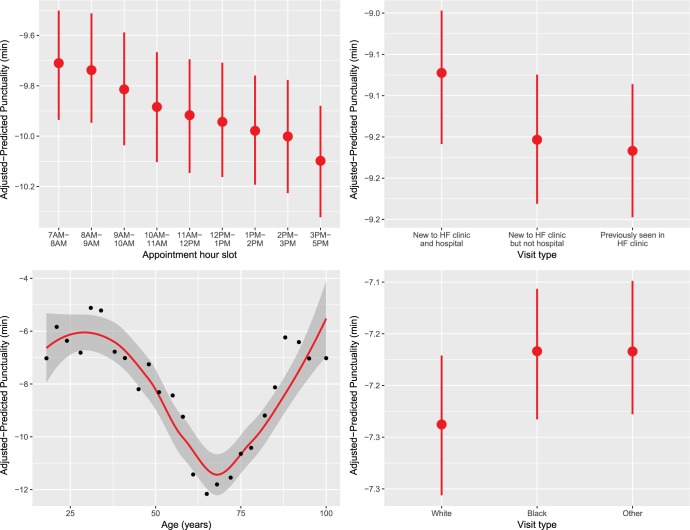
Partial dependence plots of adjusted-predicted punctuality as a function of the top 4 predictive variables identified by the minimal depth analysis. These plots were derived from the Random Forest-Regression (RF-R) machine learning framework, and can be interpreted as the effect on the response for a one unit change in the predictor, while averaging over the effects of all the other 20 variables (shown in Table 1) in the RF-R.

**Fig 4**shows partial dependence plots of the top 4 most predictive variables (from **Fig 3**) in order. Appointment hour slot, the most predictive variable, retained its linear relation with the outcome, whereby patients scheduled later in the day were predicted to check-in progressively earlier. Visit type was the second most predictive variable, whereby as compared to patients who were new to the hospital, patients who had previously visited the hospital tended to show up earlier. Age, the third most predictive variable, had a U-shaped association with the outcome, whereby the youngest and the oldest patients were predicted to show up later, as compared to those in their early 70s who were predicted to show up earliest. Race was the fourth most predictive variable, whereby patients who were white were predicted to show up earlier than those of other races.

**Fig 5:**
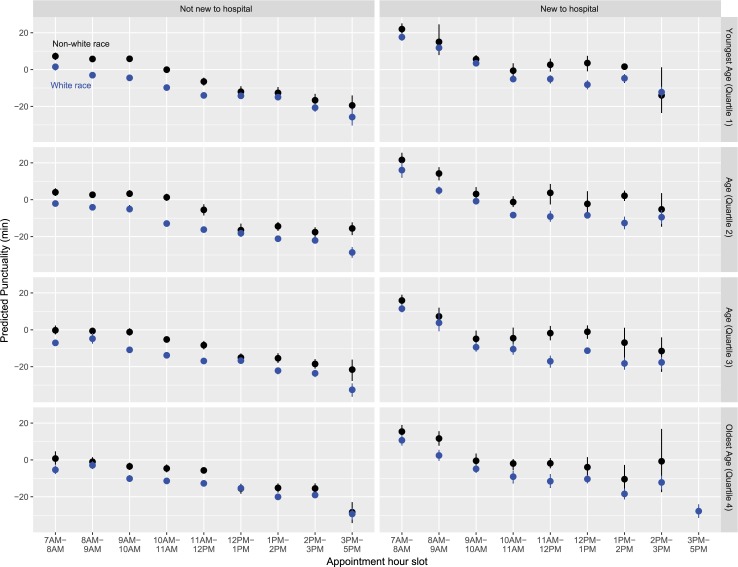
Marginal plot of predicted punctuality as a function of appointment hour slot, visit type (categories collapsed to not new versus new to hospital), age (in quartiles), and race (categories collapsed to non-white versus white race). These plots were derived from the Random Forest-Regression (RF-R) machine learning framework.

**Fig 5**is a marginal plot simultaneously visualizing the association between appointment hour slot, visit type (categories collapsed to not new versus new to hospital), age (in quartiles), and race (categories collapsed to non-white versus white race), with the outcome of predicted punctuality.

## Discussion

The main finding of our study is that on average patients attending an ambulatory outpatient HF clinic show up early prior to their scheduled visit, and not late as we initially hypothesized. Further, we found that patients tend to show up progressively earlier as the day goes on. We found that appointment hour slot and visit type were the strongest risk-adjusted predictors of arrival patterns, with other variables contributing to prediction more modestly.

We are the first to describe patterns of patient arrival to an ambulatory outpatient HF clinic. Our findings of early arrival, with progressively early arrival as the day goes on, are consistent with observations from pediatric clinics, pain clinics, and general medical clinics both in the United Kingdom and the United States [3, 13–17]. Strengths of our study include digital documentation of check-in times, large cohort size, availability of relevant adjusting variables via administrative dataset without any missing data, and a contemporary approach to data analysis using robust novel machine learning techniques.

It is not entirely clear why patients tend to arrive to outpatient appointments progressively earlier as the day goes on. We expected that this association would be attenuated by one or more other appointments or tests on the same day, but did not find it to be the case after multi-variable risk adjustment. It may be that our findings highlight an aspect of human behavior not accounted for by other variables in our models: patients want to be at home in the evenings, and they show up progressively earlier to later in the day appointments in an effort to assure this. This should be studied further in future investigations.

Our findings are timely for another reason as well. The Institute of Medicine published a statement in 2015 entitled “Transforming Health Care Scheduling and Access: Getting to Now”, which has increased concerns regarding patient wait times [1]. While most work towards reducing global wait times has focused on improving access, little attention has been paid to the role of patient arrival times as a component of this phenomenon.

An area of active investigation is the impact of wait times on the patient experience. Previously published work showed that prolonged wait times in ambulatory clinics and the emergency department negatively impacted the patient experience [2, 18–23]. This included not only reduced overall satisfaction, but also worse perceptions of information, instructions, and the overall treatment provided by various clinicians [2]. Interestingly, a different study suggested that patients who arrive late have higher satisfaction due to shorter wait times [24]. Future work should focus on whether and how the patient experience is impacted by prolonged wait times related to early arrival to outpatient clinic.

Recognition that patients tend to arrive earlier as the day goes on may further allow for providers to optimize outpatient clinic operations. Other investigators have proposed decision algorithms about whether to see an early arriving patient immediately or wait for next scheduled patient to arrive first (the “Wait-Preempt Dilemma”) [17]. Clinicians may elect to adopt such algorithms in order to reduce total patient time in clinic, optimize clinic flow, and improve the patient experience. Even simpler may be the recognition that clinicians should be physically available to start their clinic 15 to 30 minutes early for afternoon sessions, as patients are anticipated to be already waiting to be seen in this frame of time.

Our study has several limitations. First, this was a single-center experience from a large tertiary referral hospital in an urban center, which may limit generalizability. Second, we used an administrative database that lacked more detailed clinical data specific to patients with HF. It may be possible that markers of HF disease severity could be associated with punctuality, this should be investigated in future studies. Third, we did not have any detailed data about daily traffic patterns (traffic jams, parking problems, etc.) that could have been associated with punctuality.

In summary, we found an unanticipated pattern of early arrival of patients to our HF clinic, with progressively earlier arrival over the course of the day. Our results, if confirmed, have significant implications regarding estimation of patient wait times before appointment. A more nuanced understanding of punctuality in this setting may impact how clinicians and administrators design and optimize outpatient HF clinics, and the subsequent impact on workflow and the patient experience.

## Supporting information

S1 Fig CONSORT diagram. (PDF)

S1 Table Linear regression models. (DOCX)

S1 File Ambulatory HF clinic schedule templates. (DOCX)

S2 File Dataset. (CSV)
