# Supplementary figures and images for: Discordance between 'actual' and 'scheduled' check-in times at a heart failure clinic
Source: PLoS One. 2017 Nov 14;12(11):e0187849. doi: 10.1371/journal.pone.0187849 (PMC5685632; doi:10.1371/journal.pone.0187849)

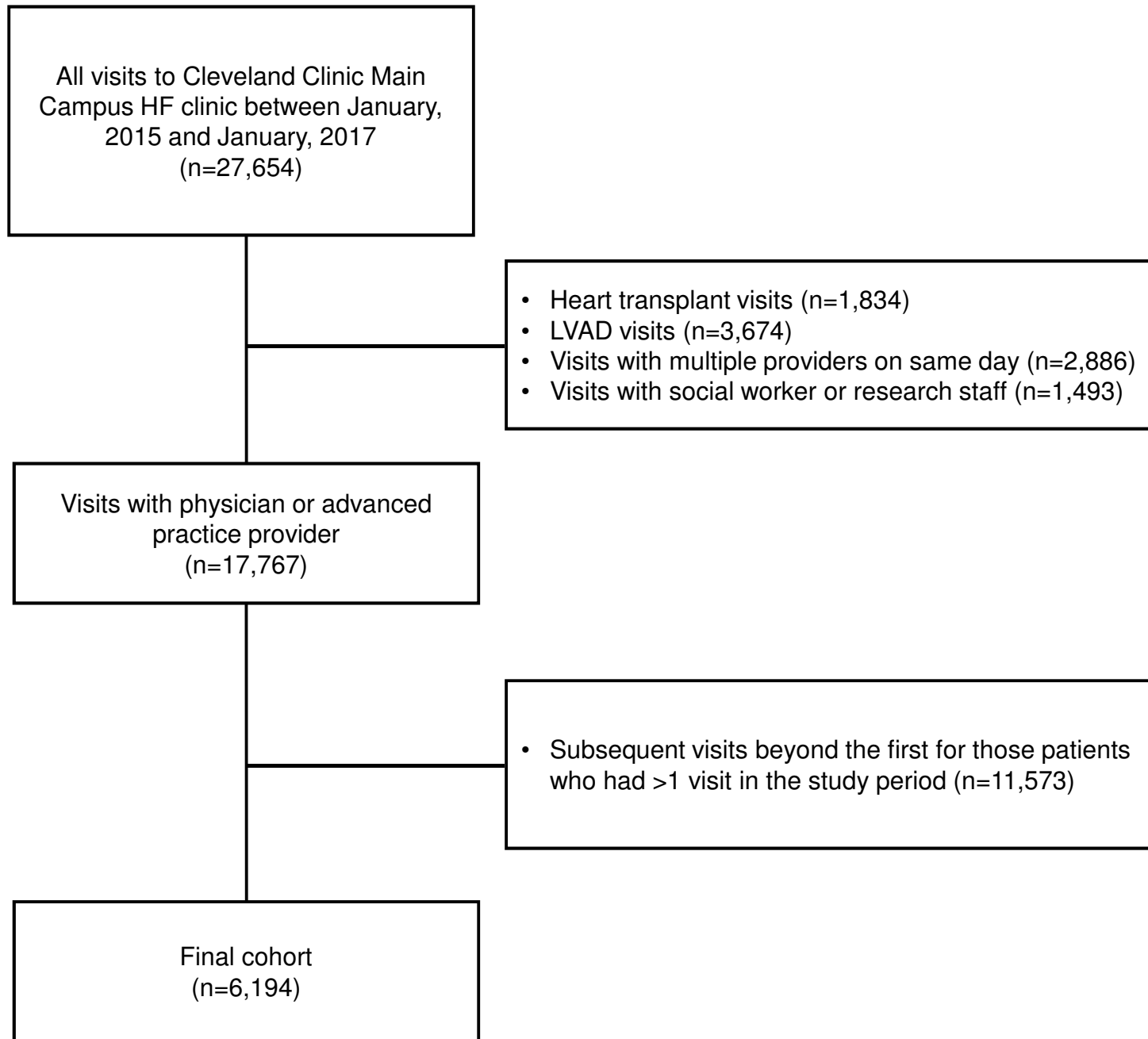

Supplement: S1 Fig — (PDF) [file pone.0187849.s001.pdf]
