# Supplementary material for: Discordance between 'actual' and 'scheduled' check-in times at a heart failure clinic
Source: PLoS One. 2017 Nov 14;12(11):e0187849. doi: 10.1371/journal.pone.0187849 (PMC5685632; doi:10.1371/journal.pone.0187849)
Supplement: S1 Table — (DOCX) [file pone.0187849.s002.docx]

|  |  | **Unpunctuality (min)** | |  |  |  |
| --- | --- | --- | --- | --- | --- | --- |
|  |  | **Β** | **SE** | **t-statistic** | **95% CI** | **P-value** |
| **Model 1** |  |  |  |  |  |  |
|  | Appointment hour slot (by 1 hour blocks) as continuous variable | -3.4 | .2 | -14.1 | (-3.8, -2.9) | <.001 |
|  |  |  |  |  |  |  |
| **Model 2** |  |  |  |  |  |  |
|  | Appointment hour slot as ordinal variable |  |  |  |  |  |
|  | 8AM-9AM vs. 7AM-8AM | -4.2 | 2.4 | -1.7 | (-9, 0.6) | .09 |
|  | 9AM-10AM vs. 7AM-8AM | -10.6 | 2.4 | -4.4 | (-15.3, -5.8) | <.001 |
|  | 10AM-11AM vs. 7AM-8AM | -13.4 | 2.4 | -5.5 | (-18.2, -8.6) | <.001 |
|  | 11AM-12PM vs. 7AM-8AM | -16.1 | 2.4 | -6.6 | (-20.8, -11.3) | <.001 |
|  | 12PM-1PM vs. 7AM-8AM | -18.3 | 2.4 | -7.6 | (-23.1, -13.6) | <.001 |
|  | 1PM-2PM vs. 7AM-8AM | -20.9 | 2.6 | -8.2 | (-26, -15.9) | <.001 |
|  | 2PM-3PM vs. 7AM-8AM | -23.8 | 2.6 | -9.3 | (-28.8, -18.8) | <.001 |
|  | 3PM-5PM vs. 7AM-8AM | -35 | 3.6 | -9.7 | (-42.1, -28) | <.001 |

Both Model 1 and Model 2 were fully adjusted for: age, sex, race, insurance, county, international, visit provider type, visit type, appointment hour slot, total outpatient appointments on same day, HF clinic visit, days of week, calendar year quarter, temperature, average wind speed, rain fall, snow fall, snow depth, fog, and haze.

**Model 1**: Multiple R-squared 6%, adjusted R-squared 5.6%, F(28, 6165) 14, P-value <.001

**Model 2**: Multiple R-squared 6.2%, adjusted R-squared 5.6%, F(35, 6158) 11.5, P-value <.001
