# Supplementary material for: Discordance between 'actual' and 'scheduled' check-in times at a heart failure clinic
Source: PLoS One. 2017 Nov 14;12(11):e0187849. doi: 10.1371/journal.pone.0187849 (PMC5685632; doi:10.1371/journal.pone.0187849)
Supplement: S1 File — (DOCX) [file pone.0187849.s003.docx]

**Physician Template A**

| **Schedule** | | | **Length (min)** | **Block type** |
| --- | --- | --- | --- | --- |
| 8:00 AM | - | 9:00 | 60 | NEW or CON |
| 9:00 | - | 9:30 | 30 | EST |
| 10:00 | - | 10:30 | 30 | EST |
| 10:30 | - | 11:30 | 60 | NEW or CON |
| 11:30 | - | 12:00 | 30 | EST |
| 12:00 PM | - | 12:30 | 30 | EST |
| 12:30 | - | 1:00 | 30 | EST |
| 1:00 | - | 2:00 | 60 | NEW or CON |
| 2:00 | - | 2:30 | 30 | EST |
| 2:30 | - | 3:00 | 30 | EST |
| 3:00 | - | 3:30 | 30 | EST |
| 3:30 | - | 4:00 | 30 | EST |

**Physician Template B**

| **Schedule** | | | **Length (min)** | **Block type** |
| --- | --- | --- | --- | --- |
| 8:00 AM | - | 8:30 | 30 | EST |
| 8:30 | - | 9:00 | 30 | EST |
| 9:00 | - | 10:00 | 60 | NEW or CON |
| 10:00 | - | 10:30 | 30 | EST |
| 10:30 | - | 11:00 | 30 | EST |
| 11:00 | - | 11:30 | 30 | EST |
| 11:30 | - | 12:30 | 60 | NEW or CON |
| 1:00 PM | - | 1:30 | 30 | EST |
| 1:30 | - | 2:00 | 30 | EST |
| 2:00 | - | 3:00 | 60 | NEW or CON |
| 3:00 | - | 3:30 | 30 | EST |
| 3:30 | - | 4:00 | 30 | EST |

NEW: New to HF clinic and hospital

CON: New to HF clinic but not hospital

EST: Previously seen in HF clinic

**Physician Template C**

| **Schedule** | | | **Length (min)** | **Block type** |
| --- | --- | --- | --- | --- |
| 8:30 AM | - | 9:00 | 30 | EST |
| 9:00 | - | 9:30 | 30 | EST |
| 9:30 | - | 10:00 | 30 | EST |
| 10:00 | - | 11:00 | 60 | NEW or CON |
| 11:00 | - | 11:30 | 30 | EST |
| 11:30 | - | 12:00 | 30 | EST |
| 12:00 | - | 1:00 | 60 | NEW or CON |
| 1:00 | - | 1:30 | 30 | EST |
| 1:30 | - | 2:00 | 30 | EST |
| 2:00 | - | 2:30 | 30 | EST |
| 2:30 | - | 3:00 | 30 | EST |
| 3:00 | - | 4:00 | 60 | NEW or CON |

**Advanced Practice Practitioner Template A**

| **Schedule** | | | **Length (min)** | **Block type** |
| --- | --- | --- | --- | --- |
| 8:00 AM | - | 9:00 | 60 | CON or EST |
| 9:00 | - | 10:00 | 60 | CON or EST |
| 10:00 | - | 11:00 | 60 | CON or EST |
| 11:00 | - | 12:00 | 60 | CON or EST |
| 12:00 | - | 1:00 | 60 | CON or EST |
| 1:00 | - | 2:00 | 60 | CON or EST |
| 2:00 | - | 3:00 | 60 | CON or EST |

**Advanced Practice Practitioner Template B**

| **Schedule** | | | **Length (min)** | **Block type** |
| --- | --- | --- | --- | --- |
| 8:30 AM | - | 9:30 | 60 | CON or EST |
| 9:30 | - | 10:30 | 60 | CON or EST |
| 10:30 | - | 11:30 | 60 | CON or EST |
| 11:30 | - | 12:30 | 60 | CON or EST |
| 12:30 | - | 1:30 | 60 | CON or EST |
| 1:30 | - | 2:30 | 60 | CON or EST |
| 2:30 | - | 3:30 | 60 | CON or EST |
